# Changes in Knee Laxity and Relaxin Receptor Isoforms Expression (RXFP1/RXFP2) in the Knee throughout Estrous Cycle Phases in Rodents

**DOI:** 10.1371/journal.pone.0160984

**Published:** 2016-08-11

**Authors:** Firouzeh Dehghan, Rahman Soori, Parvin Dehghan, Khadijeh Gholami, Sekaran Muniandy, Mohammad Ali Azarbayjani, Ashril Yusof

**Affiliations:** 1 Department of Exercise Physiology, Faculty of Physical Education and Sport Sciences, University of Tehran, Tehran, Iran; 2 Health Deputy, Babol University of Medical Sciences, Babol, Iran; 3 Department of Physiology, Faculty of Medicine, University of Malaya, Kuala Lumpur, 50603, Malaysia; 4 Department of Molecular Medicine, Faculty of Medicine, University of Malaya, Kuala Lumpur, 50603, Malaysia; 5 Department of Physical Education and Sports Sciences, Central Tehran Branch, Islamic Azad University, Tehran, Iran; 6 Department of exercise science, Sports Center, University of Malaya, Kuala Lumpur, 50603, Malaysia; Universite de Rouen, FRANCE

## Abstract

The changes in knee laxity and relaxin receptor expression at different phases of rodent estrous cycle are not known. Here, changes in the parameter were investigated in rats at different phases of the estrous cycle. Estrous cycle phases of intact female rats were determined by cytological examination of the vaginal smear. Following phase identification, blood was collected for serum hormone analyses. Knee passive range of motion (ROM) was determined by using a digital miniature goniometer. The animals were then sacrificed and patellar tendon, collateral ligaments and hamstring muscles were harvested for relaxin/insulin-like family peptide receptor 1 and 2 (RXFP1/RXFP2) analyses. Knee passive ROM was the highest at proestrus followed by diestrus and the lowest at estrus. Estrogen level was the highest at proestrus while progesterone and relaxin levels were the highest at diestrus. A strong correlation was observed between relaxin and progesterone levels. At proestrus, expression of RXFP1 and RXFP2 proteins and mRNAs were the highest at proestrus followed by diestrus and estrus. The finding shows that higher level of progesterone and relaxin in diestrus might be responsible for higher laxity of knee joint in rats.

## Introduction

The female joint laxity has been reported to be influenced by hormones. Relaxin, a polypeptide hormone produced by the corpus luteum, is known to reduce the pelvic joint laxity in guinea pigs, mice and bats during pregnancy [1]. Administration of relaxin in rats decreases the strength and organization of the periodontal ligament [2]. In human females, the serum relaxin levels were reported to correlate positively with the incidence of anterior cruciate ligament (ACL) tear, suggesting of the influence of relaxin on knee laxities [3]. The expression of relaxin receptors has been reported in humans ACL [4] and in rats, both relaxin receptor isoforms, RXFP1(relaxin family peptide receptor 1) and RXFP2 (specific ligand to insulin-like peptide 3 (INSL3) were found to be expressed in the collateral ligaments and patellar tendon [5].

There were evidences which indicate the involvement of female sex hormones in modulating the knee joint laxity. Knee joint contracture created by prolonged immobilization was slightly reduced in the pregnant ratsas compared to non-pregnant rats, suggesting that the female reproductive hormones during pregnancy play a therapeutic role [6]. A pregnancy-associated increase in the laxity of medial collateral ligament was also observed in rabbits [7]. Meanwhile, in humans, higher prevalence of back pain during pregnancy was associated with the increase in the pelvic ligament laxity under progesterone influence [8]. Administration of a physiological dose of estrogen to the ovariectomized sheep however has no effect on the ACL and medial collateral ligaments (MCL) laxities [9]. On the other hand, knee laxity in female was also found to be increased during the ovulatory or post-ovulatory phases of the menstrual cycle [10, 11]. Furthermore, knee laxity was reported to be high under the progesterone influence during the luteal phase of the menstrual cycle [12]. These findings suggested that in humans steroid hormones could cause an increase in knee laxity.

Although changes in the knee laxity have been reported throughout phases of the human menstrual cycle, the laxity change throughout the rat estrous cycle phases is unknown. Since sex-steroids were reported to modulate the expression of relaxin receptor isoforms [5], we hypothesized that changes in these isoforms expression in the knee and hamstring muscles which controls the knee joint movement might affect the knee laxity throughout phases of the estrous cycle. This study was aimed to investigate the changes in knee passive range of motion (ROM), steroid hormone levels, and the expression of relaxin receptor isoforms, RXFP1 and RXFP2 in the patellar tendon, collateral ligaments and hamstring muscles in rodents, which could explain changes in the laxity at different phases of the estrous cycle.

## Materials and Methods

### Animals

Adult WKY (Wistar-Kyoto) female rats (8–10 weeks of age, 180–200 g of weight) were provided by the Animal Experimental Unit, University of Malaya. All procedures involving animal experiments were carried out in strict accordance with the recommendations in the Guide for the Care and Use of Laboratory Animals of the United States Institute of Animal Research guidelines [13]. The protocol was approved by the Committee on the Ethics of Animal Experiments of the University of Malaya with ethics number: FIS/22/11/2011/FD(R). All surgery was performed under ketamine & xylazine (80 + 8 mg/kg) anaesthesia, and all effort was were made to minimize suffering. The animals were housed in a clean and well-ventilated standard environment of 12:12 h light: dark cycle with controlled temperature and humidity (n = 6 rats per cage). The animals had free access to soy-free diet (Gold Coin Pellet) and tap water *ad libitum*.

### Estrous phase identification

Vaginal secretions were collected by using a plastic pipette filled with 10 μL of normal saline (NaCl 0.9%). The tip of the pipette was inserted into the rat vagina, but not deeply to avoid cervical stimulation. Unstained material was placed onto a slide and was observed under a light microscope. The proportion of different cells was used to determine the estrous cycle phases, in which round and nucleated cells are the epithelial cells which define proestrus; irregular shape cells without nuclei are the cornified cells observed during estrus; little round cells are the leukocytes that characterized diestrus. At metestrus, however, three different types of cells could be identified [14].

The animals were divided into four groups based on their estrous cycle phases (proestrus, estrus, metestrus and diestrus). Following estrous cycle phases identification, the rats were anesthetized and knee passive ROM was determined using a digital miniature goniometer [15]. The blood was then collected via a heart puncture for the serum hormones analyses. Rats were then sacrificed by cervical dislocation. Knee patellar tendon, collateral ligament and hamstring muscles were harvested and placed into RNA-Later solution for real-time PCR or snapped frozen in the liquid nitrogen for Western blot analyses. The animals were divided into four groups based on their estrous cycle phases (proestrus, estrus, metestrus and diestrus).

### Serum hormone measurement

Blood samples were collected into a separator tube (SST) and were allowed to clot for 30 minutes at room temperature. The samples were centrifuged at 3000×g, for 15 minutes. Serum samples were aliquot and stored at -20°C. Radioimmunoassay (RIA) was used to determine serum levels of oestrogen in pg/ml, and progesterone in ng/ml. Serum samples were analysed in duplicate for relaxin concentration by using specific rat relaxin peptide enzyme-linked immunosorbent assay (ELISA) kit (CUSABIO—USA, Catalog Number: CSB-EL019749 RA). ELISA was performed according to the manufacturer's guidelines. The absorbance for relaxin was determined by using a microplate reader (iMark; Bio—Rad, Hercules, CA, USA) at a wavelength of 450 nm. A set of standard serial dilutions of known concentrations of relxin were provided by the manufacturer and were used to construct a standard curve in order to determine the hormone levels (150, 75, 37.5, 18.75, 9.4, 4.7, 2.4, 0 pg/ml).

### mRNA Expression Analysis by Real Time PCR (qPCR)

Real time PCR (Polymerase Chain Reaction) technique was used to calculate gene expression. This method is able to detect small difference between samples compare to other methods [16]. Patellar tendon, also known as patella ligament, which connects patella to tibia tuberosity, and lateral collateral ligament (LCL), which connects the lateral epicondyle of the femur to the head of the fibula at the lateral side of the knee joint were harvested from rat’s left hind leg. Both ligaments are extracapsular. Hamstring muscles were removed from their bony attachment. Tissues were immediately rinsed with 0.1% phosphate buffer and kept in RNA Later solution (Ambion, USA) prior to RNA extraction. Total RNA was extracted from 30 mg of tissues (wet weight) that were floating into the RNA later^®^ solution using the RNeasy Fibrous tissue Mini kit, a kit specific for fibre-rich tissues (QIAGEN, Germany). The RNA purity and concentration was assessed by 260/280 UV absorption ratios (Gene Quant 1300, UK). Two-step real time PCR was performed in two separate reactions to evaluate the gene expression.

All reagents including probes and primers were obtained from Applied Biosystems, USA. TaqMan probe (known as fluorogenic 5´ nuclease) was chosen to perform multiplex PCR. This probe has a sensitivity of 100% and a specificity of 96.67% [17] and is capable of detecting as few as 50 copies of RNA/ml and as low as 5–10 molecules [18]. Primers were designed by the same company for specific targets: RXFP1: Rn01495351; Lot no: 926762, and RXFP2: Rn01412901; Lot no: 651878, amplifies 116 bp segment of RXFP1 from the whole mRNA length of 2277 bp, and 138 bp segment of RXFP2 from the whole mRNA length of 2214 bp respectively. Hprt1 and GAPDH were used as reference genes. The catalogue number for the housekeeping genes are: GAPDH, Rn99999916_S1, Lot no: 10377343, which amplifies 87 bp segment of GAPDH from the mRNA length of 1307 bp and Hprt1, Rn01527840, Lot No: 1118680, which amplifies 67 bp, segment from the mRNA length of 1260 bp. The target assay was validated in-silico using whole rat genome sequences and in-vitro by the using whole rat cDNA sequences to ensure target sequences were detected (Applied Biosystems, USA).

All amplification experiments were done in 3 biological replicates. Amplification program include 15 minutes at 48°C (reverse transcriptase), 10 minutes at 95°C activation of ampli Taq gold DNA polymerase, denaturation at 95°C for 15 second and annealing at 60°C for 1 minute. Denaturation and annealing steps were performed for 40 cycles. Step One Plus real time PCR machine, TaqMan Fast Advanced Master Mix and assays were purchased from Applied Biosystems, USA. The assay used (TaqMan^®^-Rxfp1: Rn01495351; Lot No: 926762 and Rxfp2: Rn01412901; Lot No: 651878) amplifies a 116 bp segment of Rxfp1 from the whole mRNA length of 2277 and 138 bp segment of Rxfp2 from the whole mRNA length of 2214 bp. The catalogue number for the housekeeping genes were: Gapdh, Rn99999916_S1, Lot No: 10377343 which amplifies a 87 bp segment of Gapdh from the mRNA length of 1307 bp and Hprt1, Rn01527840, Lot No: 1118680 which amplifies a 67 bp segment from the mRNA length of 1260 bp.

Selection of stable reference genes for normalization of qPCR data analysis was performed by initial screening of six commonly used genes. Then two most stable and suitable genes (Hprt1 and GAPDH) were selected for further qPCR experiments, in which geometric means of chosen reference genes used for analysis [19]. Data was analysed according to the Comparative Ct (2^-ΔΔCt^) method [20], where amplification of the target and of the reference genes were measured in the samples and reference. Measurements were normalized using Gen Ex software. The relative quantity of target was determined by comparing the normalized target quantity in each sample to the normalized target quantity in the reference. Data Assist v3 software from Applied Biosystems, USA was used to calculate the RNA folds changes.

### Protein expression analysis by Western blotting

Patellar tendon, hamstring muscles and collateral ligaments were removed from their attachment of the rat left hind leg. These tissues were then snapped frozen in the liquid nitrogen and stored at -80°C prior to protein extraction. Total amount of protein was extracted from 50mg tissue (wet weight). Following total protein extraction with PRO-PREP (Intron, UK), equal amount of protein from each tissue lysate were mixed with a loading dye, and separated by SDS-PAGE 12%. The protein was then transferred onto a PVDF membrane (BIORAD, UK) and blocked with 5% BSA for 90 minutes at a room temperature. The membrane was exposed to rabbit polyclonal primary RXFP-1/LGR7 antibody (Abcam, UK), mouse polyclonal RXFP2/LGR8 (Abcam, UK) and rabbit anti-mouse beta actin (Abcam, UK) diluted at 1:1000 in PBS containing 1% BSA and tween-20 for 90 minutes. Blots were washed three times with each lasting for five minutes, and were then incubated with anti-rabbit or anti-mouse horseradish peroxidase conjugated secondary antibodies (Abcam, UK) at a dilution of 1:2000, for 1 hour. The membrane was then washed and subjected to Opti-4CN^™^ Substrate (Bio-Rad, USA) to visualize the protein bands. Photos of each blot were captured using a gel documentation system and density of each band was determined using Image J software. Ratio of each target band/β actin was calculated and was considered as the expression level of the target proteins.

### Antibody validation

The primary antibodies for RXFP1 and RXFP2 proteins were validated using western blot in rat tissue. Total proteins concentration of 5, 2.5, 1.25, and 0.5 μg were loaded from a grossly dissected rat tissue, measured by BCA protein assay (Thermo Fischer Scientific, Waltham, MA, USA).

### Measurement of Knee Passive ROM

Rat’s knee ROM was determined using a digital miniature goniometer [15]. Following estrous phase identification, the rats were anesthetized using ketamine and xylazine (80 + 8 mg/kg). The depth of anesthesia was confirmed from the lack of response to a painful stimuli, which was applied to the plantar surface [21]. Maintenance of animals in deep anesthesia is important as to prevent active muscle contraction in response to a painful stimuli, which will then result in the increased resistance towards the passive traction. Hip and knee joints were fixed and rested on the sensor. Meanwhile, the lower leg (knee up to ankle) was tied *in-situ* onto the device arm. Knee passive ROM was measured by pulling the device arm in a clockwise direction at a minimum constant force of 12 ± 1 Newton (N) using a mini digital Newton meter (American Weight, Loiusville, KY, USA; model: AMW-SR-1KG). The changes in value of force can be observed from the screen of the newton meter. Once the force exceeded 13 N, traction was immediately terminated and the angle obtained was recorded which represents the passive knee extension. Determination of the angle was made in different study groups. Angle was analyzed by using Torque principle. Torque is defined as the tendency of a force to rotate an object around a fixed axis which is given by “τ = r Fsinθ” formula [22]. r: rat leg length (r); F: force (A); θ: angle between the applied force and rat’s ROM.

### Statistical analysis

All data were presented as mean ± standard error of mean (SEM). Shapiro-Wilk test was applied to evaluate data normality and homogeneity distribution. One way ANOVA, with Tukey’s post-hoc test was used to determine pair wise difference, and the level of significance was set at p<0.05. Pearson and Spearman correlation coefficients were applied to determine correlation between knee range of motion and hormones levels. Density of each band in Western blot was analyzed by using Image J software, and the results were presented as the ratio of target proteins to β-actin. SPSS 18.0 statistical package was used in this study.

## Results

### Knee Passive ROM

**Fig 1:**
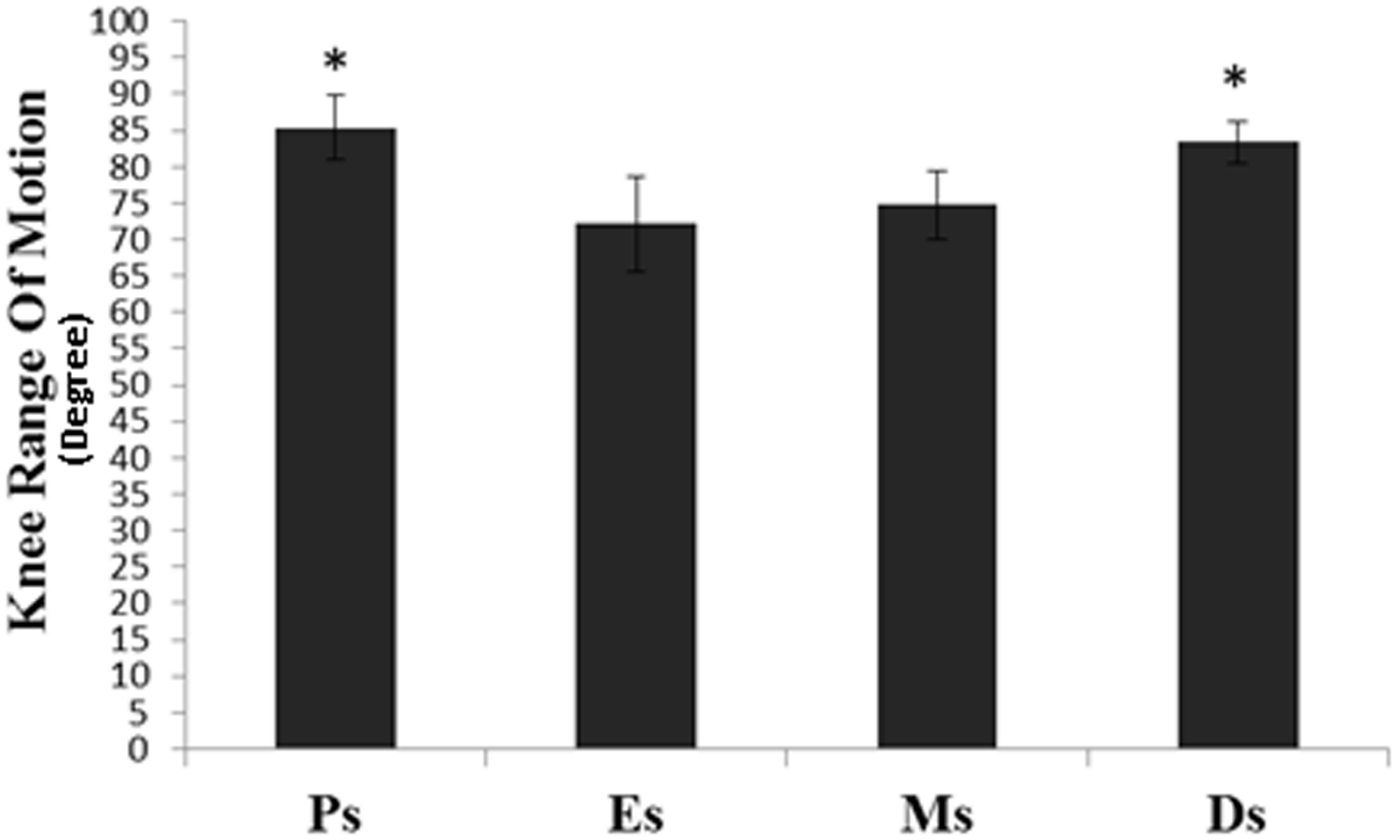
Knee passive ROM at different phases of the estrous cycle. The knee ROM was the highest at proestrus and diestrus and the lowest at estrus. Ps- proestrus; Es-estrus; Ms; metestrus; Ds; diestrus. Data were expressed as mean ± SEM and n = 6 per study group. *p<0.05 as compared to estrus.

Fig 1 shows knee passive ROM in intact female rats at different phases of estrous cycle. The passive knee ROM was significantly higher at proestrus and diestrus compared to estrus (p<0.05).

### RXFP1 & RXFP2 expressions in hamstring muscle

**Fig 2:**
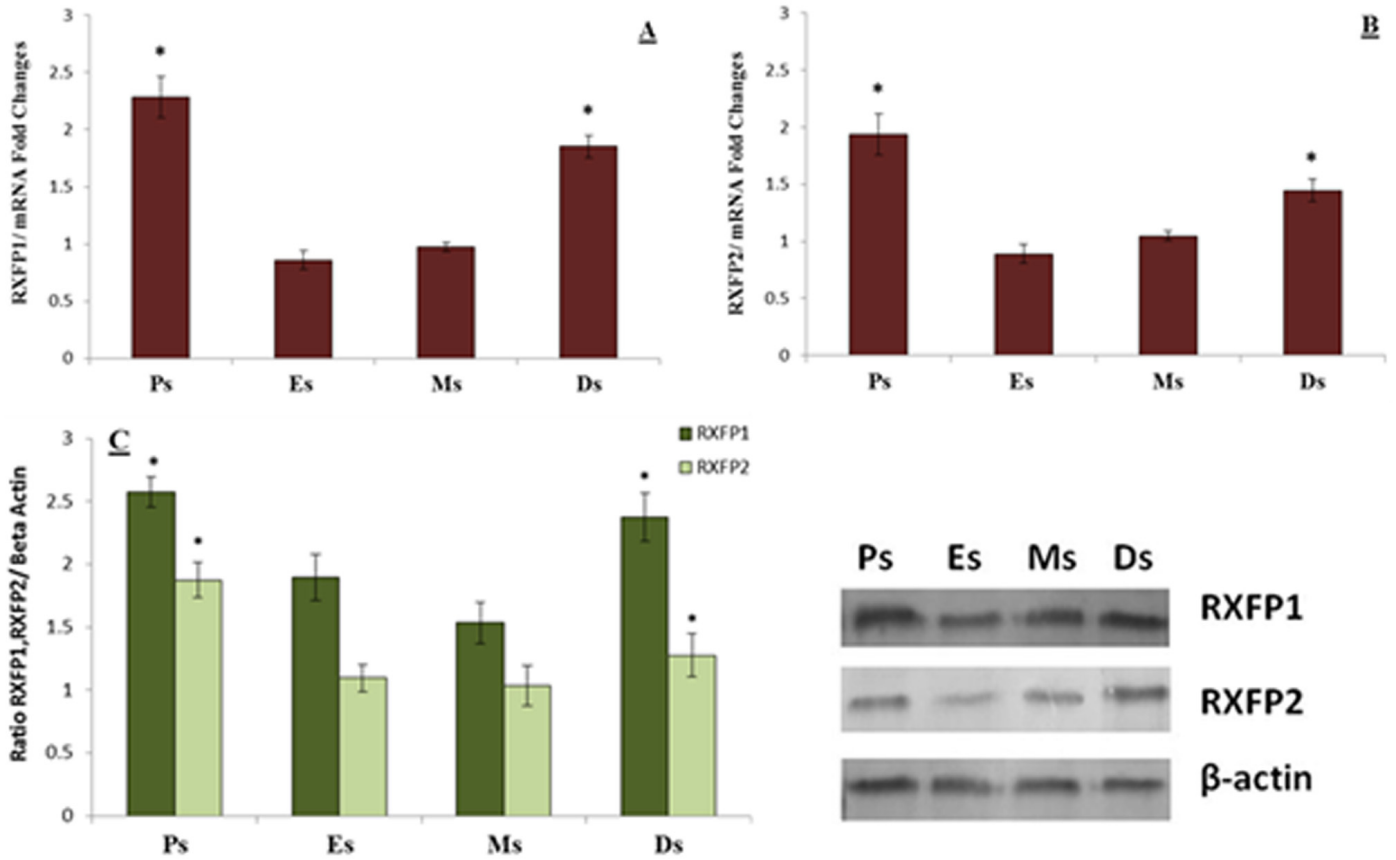
RXFP1& RXFP2 mRNA and protein expressions in the hamstring muscles. The expression of **(A)** RXFP1 mRNA **(B)** RXFP2 mRNA and **(C)** the ratio of RXFP1& RXFP2/ β-actin and Western blot images of these proteins in the hamstring muscle homogenates. The highest RXFP1 and RXFP2 mRNA and protein expressions were observed at proestrus followed by diestrus. *p<0.05 as compared to estrus for the respective isoforms. Ps-proestrus; Es-estrus; Ms; metestrus; Ds; diestrus. Data were expressed as mean ± SEM and n = 6 per study group.

Fig 2 shows (A) RXFP1 mRNA, (B) RXFP2 mRNA and (C) RXFP1 and RXFP2 over β-actin protein expression in the hamstring muscles of intact rats at different phases of the estrous cycle. Our findings indicate that RXFP1 and RXFP2 mRNA and protein levels were significantly higher at proestrus and diestrus compared to estrus.

### RXFP1 & RXFP2 expression in patellar tendon

**Fig 3:**
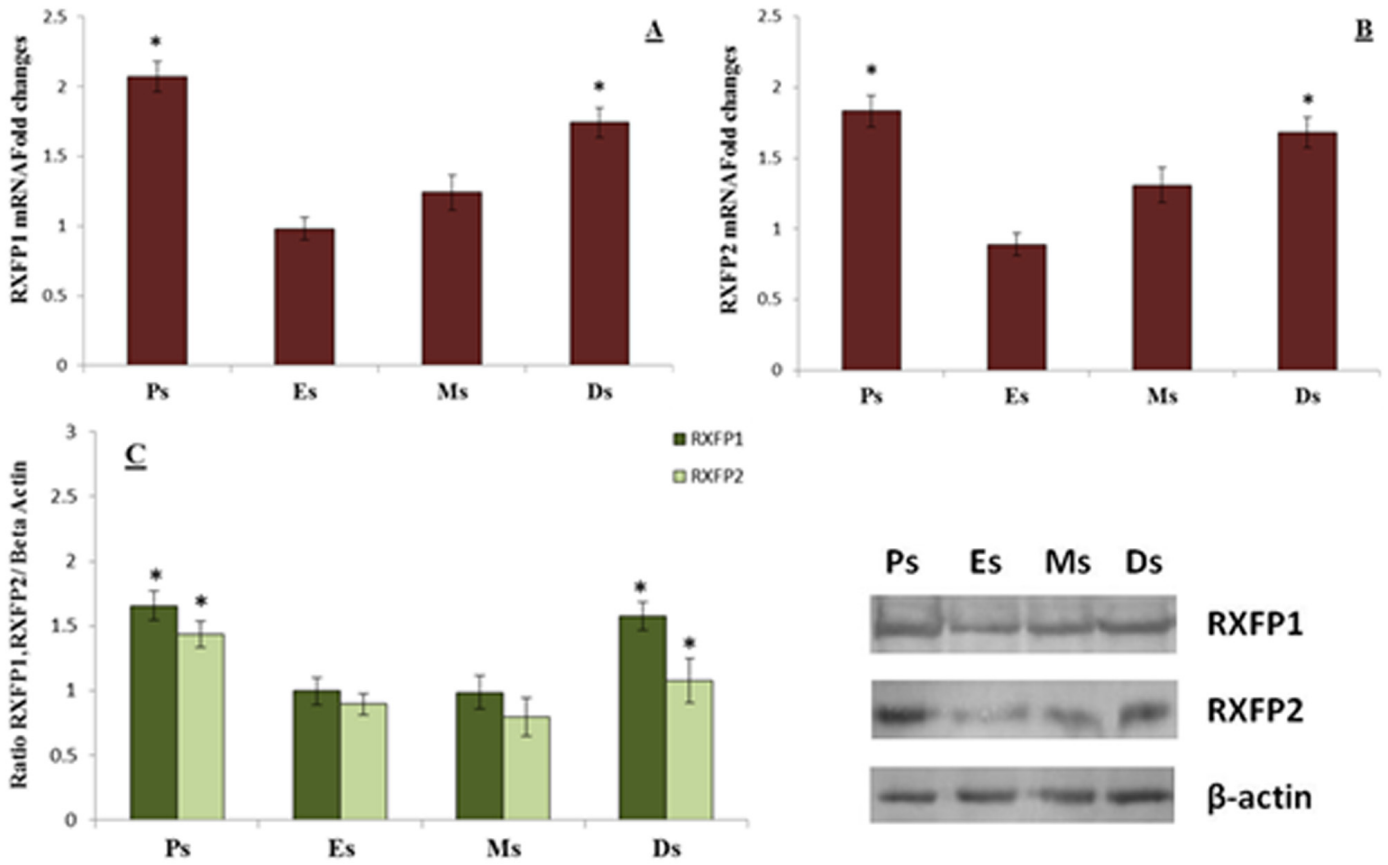
RXFP1& RXFP2 mRNA and protein expressions in the patellar tendon. The expression of **(A)** RXFP1 mRNA **(B)** RXFP2 mRNA and **(C)** ratio of RXFP1& RXFP2/ β-actin and Western blot bands of these proteins in the patellar tendon homogenates. The mRNA and protein expression were the highest at proestrus and diestrus phases. *p<0.05 as compared to estrus and metestrus for the respective isoforms. Ps-prosterous; Es-estrus; Ms; metestrus; Ds; diestrus. Data were expressed as mean ± SEM and n = 6 per study group.

Fig 3 shows (A) RXFP1 mRNA, (B) RXFP2 mRNA and (C) RXFP1 and RXFP2 over β-actin protein expression in the patellar tendon of intact rats at different phases of the estrous cycle. Our findings indicate that RXFP1 and RXFP2 mRNA and protein levels were higher at proestrus and diestrus compared to estrus. No significant differences in the expression levels of RXFP1 were noted between proestrus and diestrus phases of the estrous cycle.

### RXFP1 & RXFP2 expression in collateral ligament

**Fig 4:**
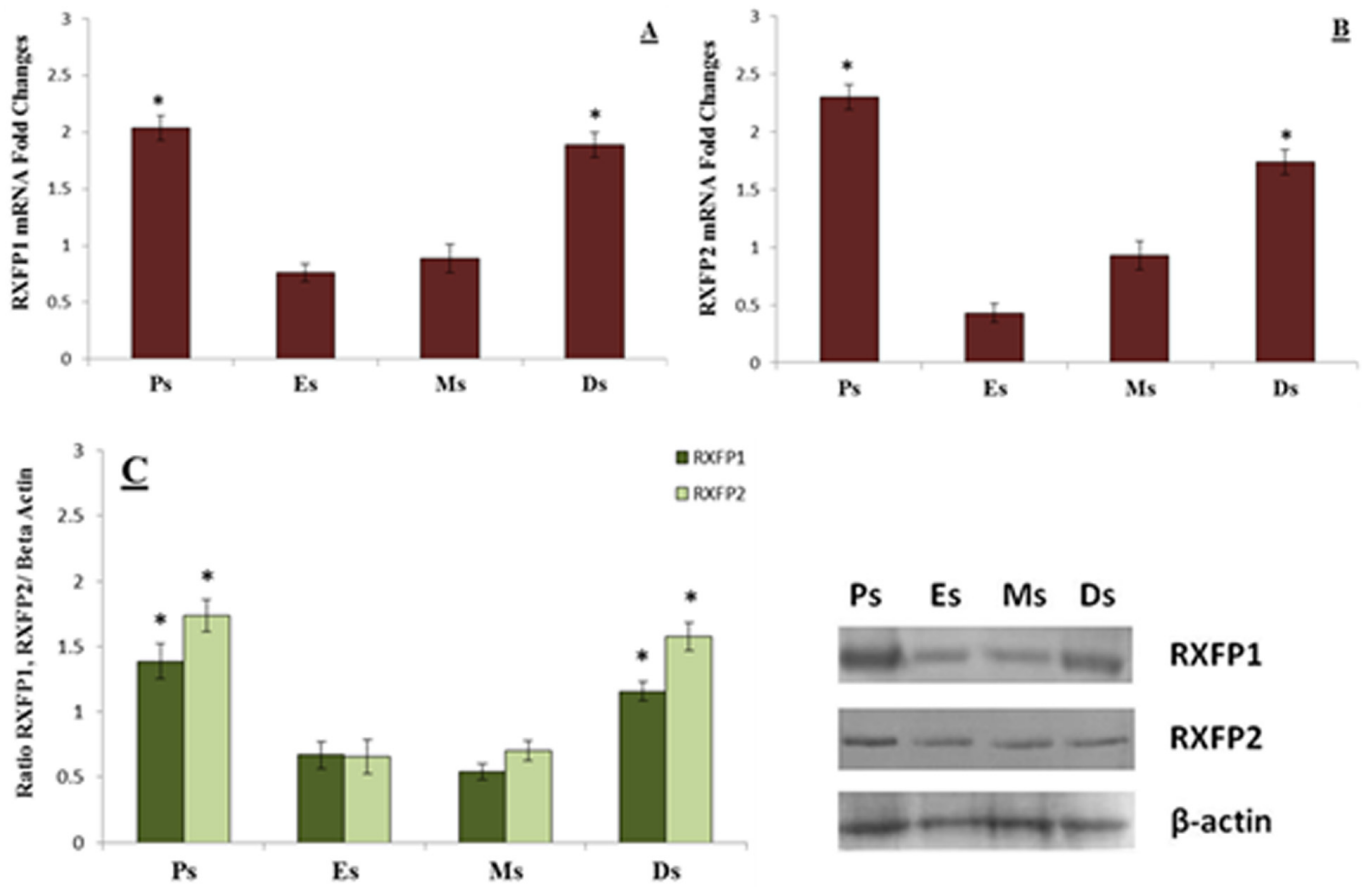
RXFP1& RXFP2 mRNA and protein expression levels in the collateral ligament. The expression of **(A)** RXFP1 mRNA **(B)** RXFP2 mRNA and **(C)** RXFP1 & RXFP2/β-actin and Western blot bands in the collateral ligament homogenates. The expression of mRNA and protein were the highest at proestrus followed by diestrus. *p<0.05 as compared to estrus and metestrus for the respective isoforms. Ps-proestrus; Es- estrus; Ms; metestrus; Ds; diestrus. Data were expressed as mean ± SEM and n = 6 per study group.

Fig 4 shows (A) RXFP1 mRNA, (B) RXFP2 mRNA and (C) RXFP1 and RXFP2 over β-actin protein expression in collateral ligaments of intact rats at different phases of estrous cycle. Our findings indicate that RXFP1 and RXFP2 mRNA and protein levels were significantly higher at proestrus and diestrus compared to estrus.

### Mean serum hormones levels in intact female rats

**Fig 5:**
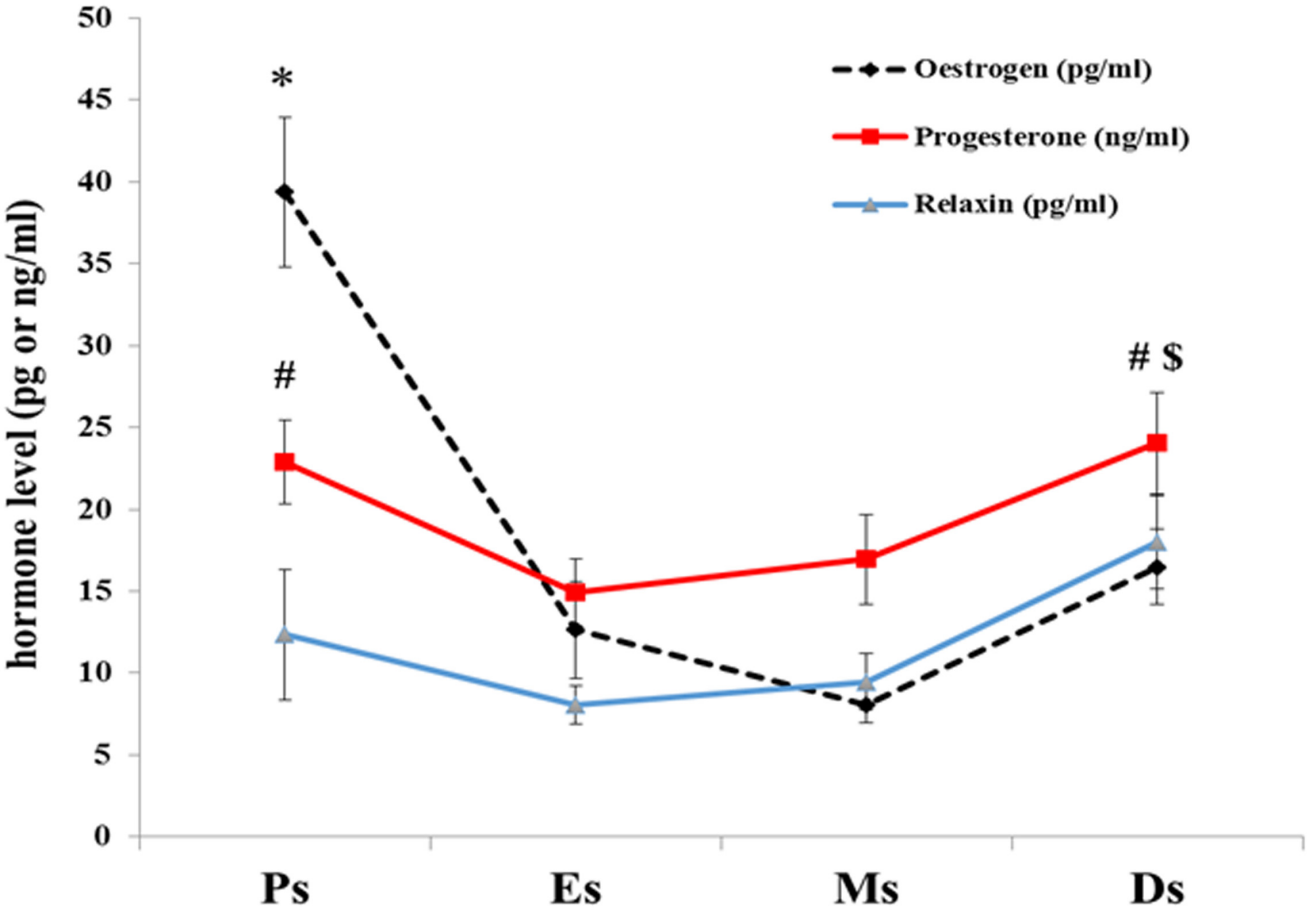
Mean serum hormone levels are presented as mean + SEM in intact female rats. *The level of oestrogen was the highest at proestrus (P<0.05). # The level of progesterone was the highest at diestrus and proestrus compare to estrus (P<0.05). $Meanwhile, relaxin level was the highest at diestrus compare to 3 other stages of estrous cycles (P<0.05). A strong positive correlation (r = 0.901 & p<001) was observed between serum progesterone and relaxin levels throughout the phases of the estrous cycle.

Table 1 shows serum levels of oestrogen, progesterone and relaxin at different phases of estrous cycle in non ovariectomized rats. The level of oestrogen was the highest at proestrus compare to 3 other stages of estrous cycles (P<0.05). The level of progesterone was the highest at diestrus compare to estrus (P<0.05), however there was no significant in progesterone level difference between proestrus and diestrus. Meanwhile, relaxin level was the highest at diestrus compare to 3 other stages of estrous cycles (P<0.05), whereas no remarkable differences observed in relaxin level at proestrus, estrus and metstrus stages (Fig 5). A strong positive correlation (r = 0.901) was observed between serum progesterone and relaxin levels throughout the phases of the estrous cycle (Fig 5).

**Table 1:** Serum hormone level in non ovariectomized rat at different phases of the estrous cycle.

Hormones	Estrous cycle	Hormone level
		(Mean ± SEM)
**Oestrogen**	Proestrus	39.38 ± 4.56 pg/ml
	Estrus	12.64 ± 2.98 pg/ml
	Metestrus	8.03 ± 1.09 pg/ml
	Diestrus	16.49 ± 2.32 pg/ml
**Progesterone**	Proestrus	22.87 ± 2.65 ng/ml
	Estrus	14.92 ± 2.05 ng/ml
	Metestrus	16.96 ± 2.73 ng/ml
	Diestrus	24.03 ± 3.08 ng/ml
**Relaxin**	Proestrus	12.34 ± 3.96 pg/ml
	Estrus	8.04 ± 1.17 pg/ml
	Metestrus	9.48 ± 1.75 pg/ml
	Diestrus	18.03 ± 2.85 pg/ml

### Validation of the Antibodies

**Fig 6:**
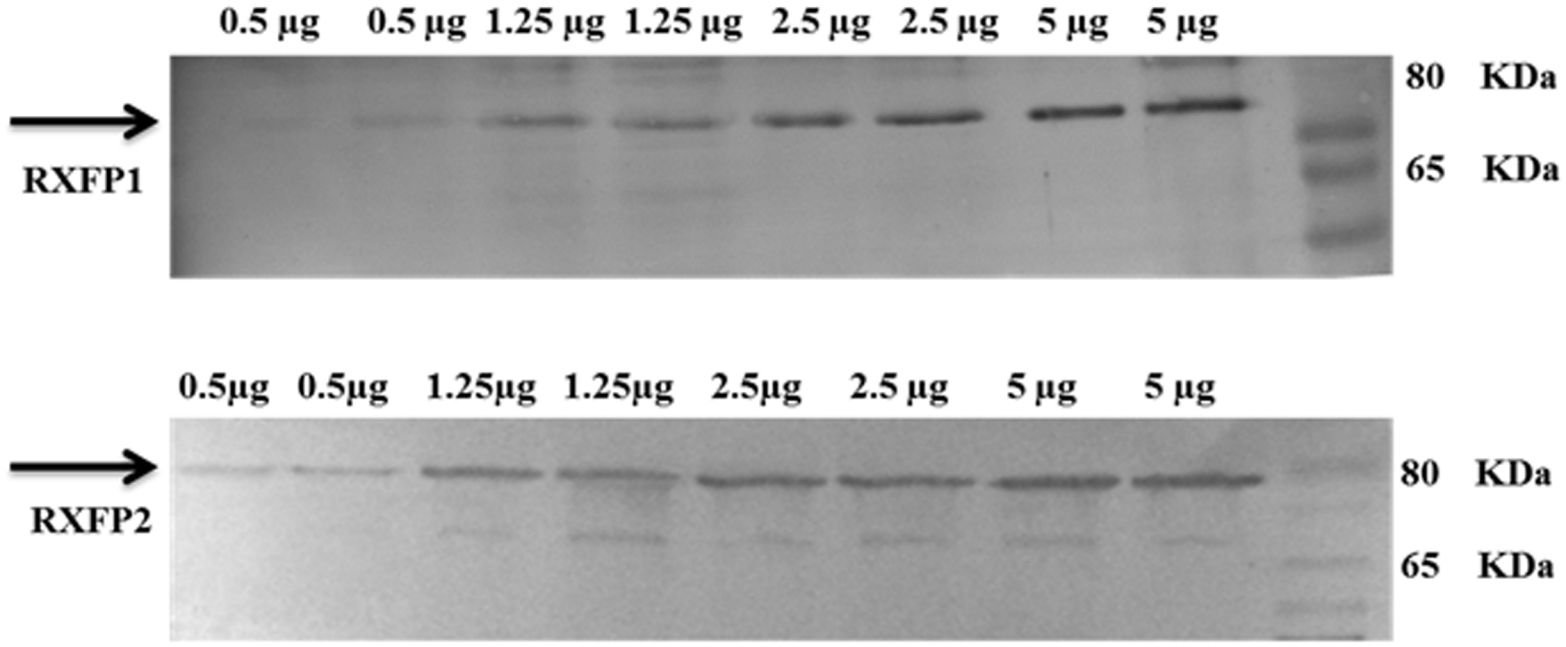
Blot analysis for validation of primary antibodies. A distinct band at ∼80 kDa, which is consistent with the molecular weight of one isoform of the RXFP1 receptor. A distinct band at ∼80 kDA is shown RXFP2 receptor, which is consistent with the molecular weight. Western blot target bands increased with increasing of sample concentration with same antibody concentration.

The result shows that the western blot target bands are increased with increasing of proteins concentration with same antibody concentration (Fig 6).

### Correlation between sex-steroid levels and knee ROM

Table 2 shows correlation between sex-steroid levels and knee ROM throughout the estrous cycle in the rats. There was no significant correlations between oestrogen and ROM whereas strong positive correlations was observed between progesterone/relaxin levels and ROM.

**Table 2:** Correlation coefficient of sex steroid and knee range of motion.

	ROM			
	Pearson		Spearman	
	Correlation	P value	Correlation	P value
**Oestrogen**	0.448	0.12	0.492	0.07
**Progesterone**	0.95	0.000***	0.74	0.001***
**Relaxin**	0.77	0.001***	0.76	0.001***

*** Correlation is significant at the 0.001 level

## Discussion

This study reports the changes in knee passive ROM in rats at different phases of the estrous cycle. Our findings indicate that the knee passive ROM was the highest at proestrus and diestrus which has the positive correlation with serum progesterone and relaxin levels. The changes in serum levels of sex-steroids during the oestrus cycle as observed in our study were consistent with the previous reports which indicate the peak oestrogen level at proestrus and the peak progesterone level occurs at diestrus [23]. The influence of female sex steroids on female knee laxity has been reported by Woodhouse et al [24] whereby the intact female rats receiving daily injection of synthetic oestrogen (ethinylestradiol) and progesterone (levonogestrel) displayed a significantly lower ACL stiffness as compared to non-treated rats during the femur-ACL-tibia loaded-to-failure test. Dragoo et al [25] reported that a combined treatment with relaxin and oestrogen to guinea pigs resulted in increased anterior tibial translation and a significantly weaker ACL on the load-to-failure test. In addition, weaker ACL was observed in relaxin administrated group. Oestrogen at increasing doses was reported to cause a decrease in the human and rabbit ACL fibroblast proliferation as well as collagen synthesis in culture [26]. In humans, reports have indicated that the high level of oestrogen during the ovulatory phase of the menstrual cycle was associated with a high incidence of ACL injury [27]. This could be due to decreased ACL laxity under the oestrogen influence [12].

Sex-steroid hormones have been reported to affect ligament laxity in different studies. However the underlying mechanism is debatable. Some studies believed that the changes in sex steroid hormones mediated knee laxity variation throughout the menstrual cycle [28–30], and might be dependent on the concentrations of hormones, but others [31, 32] refute this concept. It has been reported that in humans, ACL laxity was the lowest in the post-ovulatory (luteal) phase of the menstrual cycle [33, 34] and highest in the pre-ovulatory phase [35]. Whereas, Warden et al reported that oestrogen does not affect the mechanical properties of knee ligaments [36] and it may be more associated to neuromuscular properties through indirect effects on neuromuscular control [35, 37]. Besides endogenous steroid hormones, oral contraceptive pills (OCP) consumption may have a role to play in knee laxity. However the exact mechanism is not well known. The controversial effect of OCP may raise from different hormonal composition, pill cycle, different ligaments or tendons tissues, its influence on structure or mechanical properties [38] or does not [39–41]. Female athletes on containing high doses oestrogen and progesterone have greater ACL laxity than athletes not on OCPs [42]. The mechanisms underlying oestrogen and progesterone effect are currently not fully understood, however oestrogen has been reported to stimulate collagenase enzyme responsible for collagen breakdown [43]. Oestrogen and progesterone have been shown to up-regulate the expression of relaxin receptor isoforms, RXFP1 and RXFP2 independently in the rat’s knee [5], and that may explain the increased laxity under the influence of both hormones. However, both hormones have also been shown to inhibit the collagen synthesis in the tendon and skeletal muscles [44] which might also contribute towards a decrease in knee laxity. Therefore, variation of the reported effect of oestrogen and progesterone in human or animal model might be related to interaction between these two hormones.

Our findings have shown that the serum levels of relaxin were the highest at diestrus which were consistent with the reported increase in relaxin synthesis by the corpus luteum formed after ovulation and was maintained throughout diestrus phase of the estrous cycle [45]. In species such as rodents, relaxin plays an important role in modulating joint laxity [1]. However, in humans, its role is still debatable. The latter was supported by an observation, where a relationship was observed between peripheral joint laxity and serum relaxin levels in the females during the pregnancy period [46]. Although relaxin might not play important role in determining the ligament laxity in humans, the expression of relaxin receptors has been reported in human ACL [4] and human ACL fibroblast [47]. In rats however, relaxin most likely plays an important role in modulating knee laxity in view that its receptor expression has been reported both in the patellar tendon and collateral ligaments [15]. Additionally, relaxin has been reported to stimulate the activity of matrix metalloproteinases (MMPs) through induction of collagenase-1 and stromelysin-1, which are involved in the collagen breakdown [48, 49]. We postulated that the highest level of relaxin and progesterone during diestrus phase and its moderately high level during proestrus phase might contribute towards the increase in knee passive ROM in rats. With regards to the role of progesterone in knee passive ROM in our previous study [50], which was suppressed by its antagonist (mifepristone), so progesterone may act in parallel with relaxin since this is positive correlation between these two hormones throughout phases of the estrous cycle.

Our findings have further shown the changes in the expression of two main relaxin receptor isoforms, RXFP1 and RXFP2 in the hamstring muscles, patellar tendon and collateral ligaments which could affect knee laxity. The expression of RXFP1 and RXFP2 were the highest during proestrus followed by diestrus phase. In the hamstring muscles and patellar tendon, RXFP1 is the main isoform while in the collateral ligament, RXFP2 expression exceeds RXFP1. The up-regulation of both relaxin receptor isoforms may contribute towards increased in knee laxity both at proestrus and diestrus phases of the cycle. Changes in these isoforms expression in the hamstring muscle is important as this could affect knee extension.

## Conclusion

To summary, we have demonstrated that changes in knee ROM in rats is fluctuates with the ostrous cycle. Furthermore, the increased knee laxity at proestrus and diestrus is correlated with the high levels of serum progesterone, relaxin, and relaxin receptors expression in the rat knee tissues. The results can be implicated to understand the non-traumatic knee injury associated with the level of sex-steroid hormones. In view of this, more studies are warranted to investigate changes in knee laxity in relation to combination of sex steroid hormones or individual in cellular and molecular levels with respect to different tissues, gender, subjects.
